# Mosquito host-seeking diel rhythm and chemosensory gene expression is affected by age and *Plasmodium* stages

**DOI:** 10.1038/s41598-022-23529-7

**Published:** 2022-11-05

**Authors:** Melika Hajkazemian, Sharon R. Hill, Raimondas Mozūraitis, Lisa Ranford-Cartwright, S. Noushin Emami, Rickard Ignell

**Affiliations:** 1 Department of Molecular Biosciences, Wenner-Gren Institute, Stockholm University, Stockholm, Sweden; 2 Disease Vector Group, Department of Plant Protection Biology, SLU, Alnarp, Sweden; 3 Laboratory of Chemical and Behavioural Ecology, Institute of Ecology, Nature Research Centre, Vilnius, Lithuania; 4 Department of Zoology, Stockholm University, Stockholm, Sweden; 5 Institute of Biodiversity, Animal Health and Comparative Medicine, College of Medical, Veterinary and Life Science, University of Glasgow, Glasgow, UK; 6 Molecular Attraction AB, Elektravägen 10, Hägersten, 126 30 Stockholm, Sweden; 7 Natural Resources Institute, FES, University of Greenwich, London, UK

**Keywords:** Computational biology and bioinformatics, Molecular biology, Systems biology, Diseases

## Abstract

Malaria parasites can affect vector-related behaviours, increasing transmission success. Using *Anopheles gambiae* and *Plasmodium falciparum*, we consider the effect of interaction between infection stage and vector age on diel locomotion in response to human odour and the expression of antennal chemosensory genes. We identified age-dependent behavioural diel compartmentalisation by uninfected females post-blood meal. Infection disrupts overall and diel activity patterns compared with age-matched controls. In this study, mosquitoes carrying transmissible sporozoites were more active, shifting activity periods which corresponded with human host availability, in response to human odour. Older, uninfected, blood-fed females displayed reduced activity during their peak host-seeking period in response to human odour. Age- and infection stage-specific changes in odour-mediated locomotion coincide with altered transcript abundance of select chemosensory genes suggesting a possible molecular mechanism regulating the behaviour. We hypothesize that vector-related behaviours of female mosquitoes are altered by infection stage and further modulated by the age post-blood meal of the vector. Findings may have important implications for malaria transmission and disease dynamics.

## Introduction

Malaria parasites depend on mosquito vectors for transmission from one host to another. Increasing empirical evidence demonstrates that insect-borne pathogens may manipulate both their human host and their insect vector to enhance transmission success^1–4^. This manipulation is not static, as vector-related behaviours can vary significantly between uninfected and infected vectors over different time scales relevant to the disease transmission dynamics^3–5^. Pathogens are known to affect vector behaviours, including locomotion, flight, host seeking, probing and feeding, which can manifest in increased avidity to locate and obtain a blood meal, or in alterations in host preference, resulting in an enhanced transmission through the increased contact between vector and host^3,5,6^. The molecular mechanisms by which these behavioural traits may be modulated have only recently received attention, identifying pathways in neurosignalling and in the neuroendocrine circuitry as key targets^2,5,7,8^.

Mosquitoes acquire *Plasmodium* parasites, the causative agent of malaria, through a blood meal from an infected vertebrate host. The pathogen undergoes several developmental stages in the mosquito, from midgut invasion and oocyst formation (midgut oocyst-infected mosquitoes, 7 days post-infection; dpi) through to the invasion of the salivary glands, when a mosquito can transmit the malaria sporozoites (salivary gland sporozoite-infectious mosquitoes, 14 dpi)^9^. The avidity of mosquitoes to engage in host seeking and blood feeding behaviours, depends not only on the infection status of the mosquito, but also on the developmental stage of the pathogen in the mosquito^3^. Available data for *Plasmodium* parasites that cause avian, rodent and human malaria emphasise that vector-related behaviours, including feeding, *e.g.*, probing, persistence and feeding rate, as well as foraging, e.g., host responsiveness and location, are suppressed when mosquitoes are midgut oocyst-infected (7 dpi), but not able to transmit the pathogen, whereas salivary gland sporozoite-infectious mosquitoes are more active (14 dpi)^10–12^. This modulation appears to be conserved across vectors and other pathogens, e.g., *Aedes aegypti,* which transmit dengue and Zika virus^5,13^. The observed changes in the odour-mediated host-seeking behaviour, demonstrated for both midgut oocyst-infected and salivary gland sporozoite-infectious mosquitoes, are reflected in the selective sensitivity of the peripheral olfactory system to key host-related volatile organic compounds^5,10,14^. Such modulation may be regulated through alterations in neural signalling and chemosensory gene expression, as demonstrated in malaria- and dengue-infected mosquitoes^2,5^.

In depth analysis of the molecular basis of olfaction in mosquitoes, has allowed for the identification and functional characterisation of chemosensory gene families, including odorant receptors (*Or*s), gustatory receptors (*Gr*s), ionotropic receptors (*Ir*s), odorant binding proteins (*Obp*s) and chemosensory proteins (*Csp*s)^15–19^. The expression of select members of these gene families in olfactory-related tissues change in response to adult maturation, age and physiological state^20–25^, including infection status^5^. These state-dependent changes in gene abundance coincide with concerted alterations in the sensitivity of olfactory sensory neurones and the behaviour of the mosquitoes^20,21,25^.

This study analysed the stage-dependent effect of *P. falciparum* infection on the locomotion activity of female *Anopheles gambiae* in the presence and absence of human odour, demonstrating that midgut oocyst-infected and salivary gland sporozoite-infectious mosquitoes display differential locomotor activity correlating with age post-blood meal and time of day. The potential molecular mechanisms, underlying the age- and infection-dependent odour-mediated locomotor activity, were assessed by transcriptome analysis, identifying differentially abundant chemosensory-related genes in the antennae coinciding with the demonstrated differences in mosquito behaviour. A better understanding of the effect of infection on locomotor activity and odour-mediated host seeking throughout the day has the potential to more accurately direct vector measures. For example, if we know when salivary gland sporozoite-infectious mosquitoes are active and host seeking, especially as these time periods differ from those of uninfected females, we can develop strategies to increase the probability of protecting people from these mosquitoes posing the greatest risk to human health, such as informing the public to take special precautions (e.g., remaining under LLINs, wearing protective clothing) during these times. Identifying the molecular mechanisms by which these behavioural traits are modulated by *P. falciparum* may lead to a better understanding of vectorial capacity and transmission dynamics potentially inspiring the development of innovative control strategies.

## Results

### Mosquito age and the stage of *Plasmodium falciparum* infection affect locomotor activity

**Figure 1:**
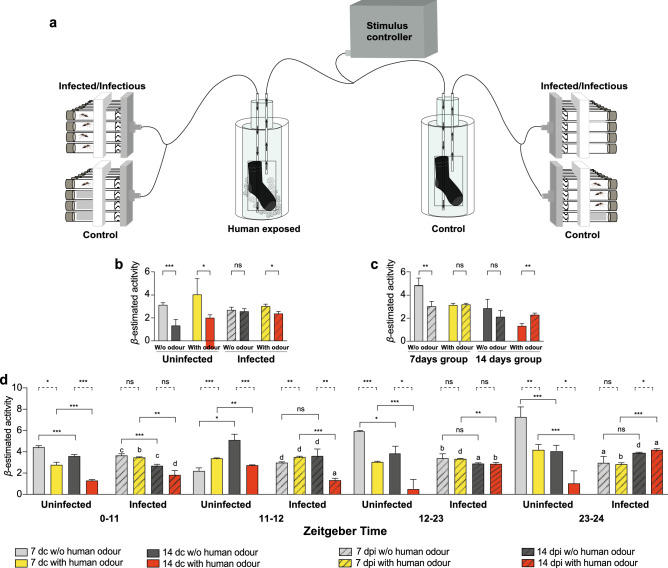
HYPERLINK "sps:id::fig1||locator::gr1||MediaObject::0"*Plasmodium falciparum* infection stage differentially affects mosquito locomotor activity. Schematic design of the locomotor assay (**a**) used to assess the activity of female *Anopheles gambiae*, 7 days post-infection (dpi) and 14 dpi with *P. falciparum* compared to age-matched uninfected females (days control, dc), in the presence (with human odour) or absence of human odour (w/o human odour). Individual locomotor profiles (n = 384), assessed for variation using a redundancy analysis, demonstrated a differential effect of age post-blood meal (the mixed model was build based on the effect of age as a main fixed variable combined with the random effect of the other variables; *β*-lmer ± SE = age + experimental replication|1); (**b**) infection (the model demonstrated the effect of the main fixed variable of infection, including the rest of the variables as random variables ; *β*-lmer ± SE = infection + experimental replication|1); and (**c**) on locomotor activity in the presence and absence of human odour. (**d**) Diel locomotor activity profiles of 7 and 14 dpi females, along with age-matched controls, demonstrated effects of both age post-blood meal, and infection in the presence and absence of human odour during informative activity periods (photophase, Zeitgeber Time (ZT) 0–11; dusk, ZT 11–12; scotophase, ZT 12–23; and dawn, ZT 23–34) over a 24 h period (the mixed model estimated the effect of all four main fixed variables including age, infection, time, human odour, as well as the random effect of the experimental replication combined with the interactions among all these variables; *β*-lmer ± SE = age + infection + time + human odour + experimental replication|1). Significant effects were determined using GLMM models (lmer). Bars represented by *β*-estimation generated by the mixed model ± SE (*β*-lmer ± SE); asterisks denote significant differences (*p < 0.05; **p < 0.01; ***p < 0.001; ns, non-significant)*.* Letters indicate the pairwise comparisons between infected and uninfected groups; a = *p* < 0.001, b = *p* < 0.01, c = *p* < 0.05, d = non-significant).

Locomotor activity of uninfected female *An. gambiae* in the absence of human odour (Supplementary Figs. 1a and 1c) significantly declined with age post-blood meal, when assessed in *Drosophila* activity monitors (χ^2^_1_ = 5.86, *p* = 0.02; Fig. 1a,b). This age effect was not observed following infection with *P. falciparum* (χ^2^_1_ = 0.83 *p* = 0.35; Fig. 1b; Supplementary Figs. 1a and 2a). In the presence of human odour, the age-dependent reduction in locomotor activity was observed in both uninfected (χ^2^_1_ = 21.12, *p* < 0.001) and *P. falciparum* carrying mosquitoes (χ^2^_1_ = 5.73, *p* = 0.02; the GLMM:lmer model was constrained by age and the random effect of experimental replication; Fig. 1b; Supplementary Figs. 1b and 2b). The infection load increased with age post-blood meal and was consistent across replicates (Supplementary Fig. 2).

Infection status significantly affected the activity of mosquitoes, when comparing the overall locomotion profiles of 7 dpi and 14 dpi individuals with age-matched uninfected females, in the presence and absence of human odour (*β*-estimated = weighted mean value based on all variables plus the random effect of the replication ± SE; presence of human odour: χ^2^_1_ = 18.06, *p* < 0.001; absence of human odour: χ^2^_1_ = 5.73, *p* < 0.01). In the absence of human odour, 7 dpi females were less active compared to age-matched controls (χ^2^_1_ = 8.21, *p* = 0.004; Supplementary Fig. 1), whereas 14 dpi females were as active as their age-matched controls (χ^2^_1_ = 2.27, *p* = 0.13; Fig. 1c; Supplementary Fig. 1c). A continuous temporal activity analysis, which provides a sliding moving average of a single variable, i.e., locomotion (Supplementary Fig. 1), supported the overall GLMM statistical model, which combines multiple variables, as well as their interaction (Fig. 1), and the overall finding of this experiment. However, the continuous temporal activity analysis also identified a discrepancy, in which 14 dc females appeared to be more active than 14 dpi females during scotophase (Supplementary Fig. 1a,c). This emphasises the statistical power of using multi-variable models for studying complex ecological interactions. The presence of human odour differentially altered the locomotor activity. While there were no significant differences between 7 dpi and age-matched females in the presence of human odour (χ^2^_1_ = 0.12, *p* = 0.73; Fig. 1c; Supplementary Fig. 1b), the salivary gland sporozoite-infectious 14 dpi mosquitoes were more active than age-matched uninfected females (14 days control; 14 dc χ^2^_1_ = 8.80, *p* = 0.002; Fig. 1c; Supplementary Fig. 1d).

### Mosquito locomotor activity varies temporally in response to host odour and *P. falciparum* infection

To assess the diurnal effect on the locomotor activity of mosquitoes^26^ with *P. falciparum* infection and their respective controls, a mixed model analysis was used (lmer model: constrained by two main explanatory variables, infection status and period of time, as well as the random effect of replication; Supplementary Tables 1 and 2). In the absence of human odour, the diurnal locomotor activity profile of uninfected mosquitoes confirmed the high activity at dawn (ZT 23–24), with the younger cohort being more active during scotophase with a peak at dawn (Supplementary Fig. 1a), whereas the older control females displayed an increased activity at dusk and scotophase (ZT 0–11: χ^2^_1_ = 27.43, *p* < 0.001; ZT 11–12: χ^2^_1_ = 4.58, *p* = 0.049; ZT 12–23: χ^2^_1_ = 4.33, *p* = 0.03; ZT 23–24: χ^2^_1_ = 32.32, *p* < 0.001, Fig. 1d, Supplementary Table 2; Supplementary Fig. 1c). In the presence of human odour, the locomotor activity of the younger uninfected cohort increased at dawn, and remained at this activity level until dusk (Supplementary Fig. 1b), while the older females only displayed an increase in activity at dusk (ZT 0–11: χ^2^_1_ = 17.54, *p* < 0.001; ZT 11–12: χ^2^_1_ = 7.67, *p* = 0.005; ZT 12–23: χ^2^_1_ = 15.21, *p* < 0.001; ZT 23–24: χ^2^_1_ = 18.25, *p* < 0.001, Fig. 1d, Supplementary Table 2; Supplementary Fig. 1d).

The time-of-day, and the stage of *P. falciparum* sporogony, differentially affected the locomotor activity of the vector (Fig. 1d; Supplementary Fig. 1). In mosquitoes with parasites undergoing sporogony, there was no effect of age post-blood meal on locomotion throughout the 24 h period in the absence of human odour (ZT 11–12: χ^2^_1_ = 1.71, *p* = 0.18; ZT 12–23: χ^2^_1_ = 0.31, *p* = 0.57; ZT 23–24: χ^2^_1_ = 3.49, *p* = 0.06, Supplementary Table 2), except during photophase, in which salivary gland sporozoite-infectious (14 dpi) mosquitoes demonstrated lower activity than midgut oocyst-infected (7 dpi) individuals (ZT 0–11: χ^2^_1_ = 21.97, *p* < 0.001). However, in the presence of human odour, the salivary gland sporozoite-infectious cohort (14 dpi) was more active at dawn (ZT 23–24: χ^2^_1_ = 15.40, *p* < 0.001; Supplementary Fig. 1d), while being less active than the younger mosquitoes throughout the rest of the diurnal period (ZT 0–11: χ^2^_1_ = 9.35, *p* = 0.002; ZT 11–12: χ^2^_1_ = 20.02, *p* < 0.001; ZT 12–23: χ^2^_1_ = 9.73, *p* = 0.001, Supplementary Table 2; Supplementary Fig. 1b). While the continuous temporal activity analysis supports the overall GLMM statistical model, and the overall finding of this experiment, it also identified a discrepancy, in which 14 dpi females appeared to be more active than 7 dpi females throughout scotophase (Supplementary Fig. 1b,d).

Individual locomotor profiles of mosquitoes at 7 dpi and 14 dpi differed significantly from that of age-matched uninfected controls (Fig. 1d). Infection significantly reduced the locomotor activity in 7 dpi females in the absence of human odour when compared with their uninfected counterparts during most time periods (ZT 0–11: χ^2^_1_ = 8.49, *p* = 0.03; ZT 11–12: χ^2^_1_ = 1.008, *p* = 0.31; ZT 12–23: χ^2^_1_ = 7.84, *p* = 0.005; ZT 23–24: χ^2^_1_ = 13.78, *p* < 0.001, Supplementary Tables 1 and 2; Supplementary Fig. 1a). Mosquitoes carrying the transmissible stage of the parasite (14 dpi) displayed a lower locomotor activity than the uninfected counterparts throughout photophase and scotophase (ZT 0–11: χ^2^_1_ = 5.92, *p* = 0.01; ZT 12–23: χ^2^_1_ = 11.87, *p* < 0.001, Supplementary Tables 1 and 2; Supplementary Fig. 1c), but not at dusk and dawn (ZT 11–12: χ^2^_1_ = 2.68, *p* = 0.10; ZT 23–24: χ^2^_1_ = 2.20, *p* = 0.10; Supplementary Fig. 1c). In the presence of human odour, the midgut oocyst-infected (7 dpi) females showed lower activity than age-matched controls at dawn (ZT 23–24: χ^2^_1_ = 7.29, *p* = 0.006 Supplementary Fig. 1b), while the inverse activity pattern was observed during photophase (ZT 0–11: χ^2^_1_ = 12.28, *p* = 0.001; Supplementary Fig. 1b). The salivary gland sporozoite-infectious mosquitoes (14 dpi) exhibited a significantly increased locomotor activity at dawn (ZT 23–24: χ^2^_1_ = 23.78, *p* < 0.001) and during scotophase (ZT 12–23: χ^2^_1_ = 25.42, *p* = 0.001), while there was no difference in activity of the salivary gland sporozoite-infectious (14 dpi) and uninfected females during photophase (ZT 0–11: χ^2^_1_ = 2.26, *p* = 0.13), with a significant reduction in dusk (ZT 11–12: χ^2^_1_ = 16.06, *p* < 0.001, Supplementary Tables 1 and 2; Supplementary Fig. 1d).

### *Plasmodium falciparum* modulates antennal transcript abundance

Paired-end sequencing of each of the libraries constructed from antennal RNA, with a total of 2400 antennae, generated an average mapping of 26,399,740 million cleaned reads per library. Out of the 13,832 coding genes annotated in the genome of *An. gambiae* (Agam4.10), a total of 10,115 transcripts were reliably detected above 1 transcript per million (TPM) mapped reads in the antennae, among all experimental and control groups, demonstrating an adequate level of coverage.

**Figure 2:**
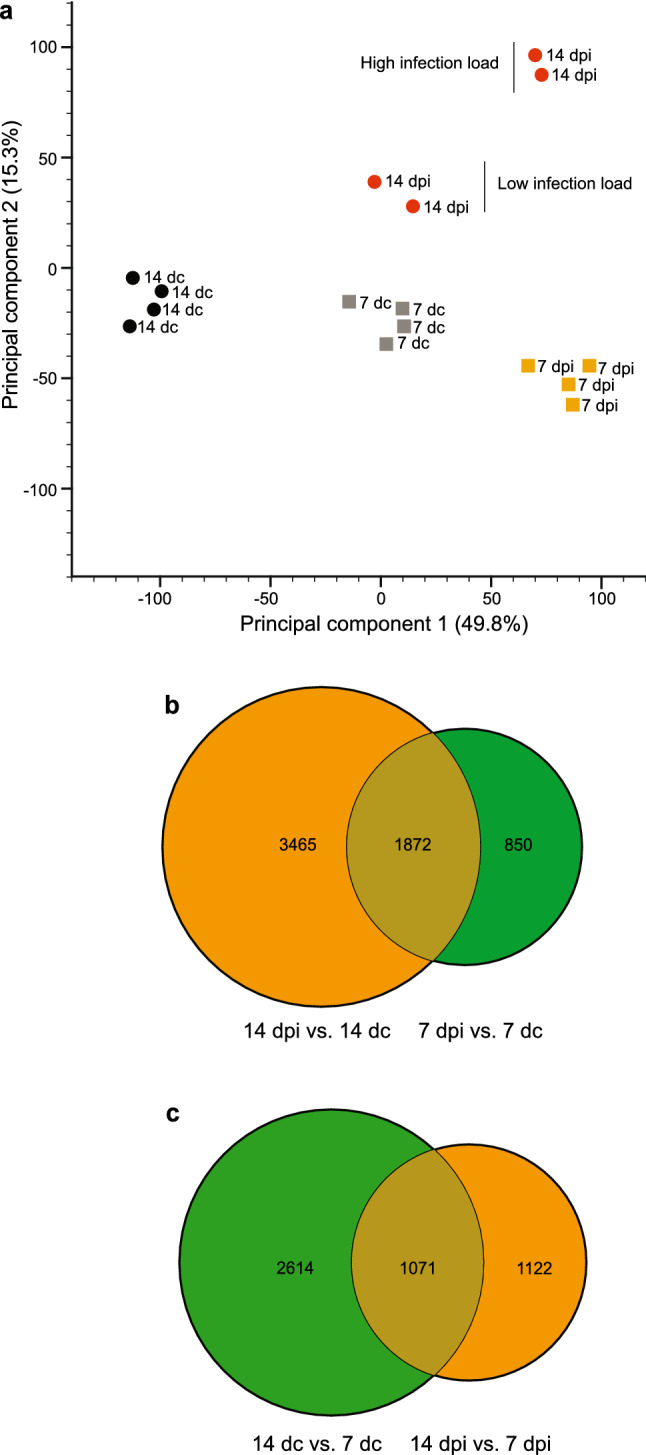
*Plasmodium falciparum* infection stage affects antennal transcript abundance in *Anopheles gambiae*. Principal component analysis (**a**) of the overall transcript abundance in the antennae of midgut oocyst-infected (7 days post-infection, dpi) and salivary gland sporozoite-infectious (14 dpi) mosquitoes compared to age-matched uninfected mosquitoes (7 days control, dc and 14 dc). *Plasmodium falciparum* infection load is indicated (see Supplementary Fig. 1). Proportional Venn diagrams (**b**, **c**) depicting pairwise comparisons among the antennal transcriptomes of midgut oocyst-infected, salivary gland sporozoite-infectious, and age-matched uninfected females. Overlapping regions represent the subsets of transcripts that are shared between the different conditions. Significant differences in transcript abundance were determined as a fold change of greater than 1.5, and an FDR-corrected *p*-value < 0.05.

A principal component analysis of the antennal transcripts was conducted to demonstrate the overall variation among the antennal transcriptomes (midgut oocyst-infected (7 dpi), salivary gland sporozoite-infectious (14 dpi), and age-matched uninfected conditions; 4 replicates each; Fig. 2a). The principal component analysis identified that 49.8% of the variation among the libraries was based on the relative infection status in each age group (principal component 1; PC1), while 15.3% of the variance was dependent on age post-blood meal and infection status relative to the controls (PC2; Fig. 2a). All of the biological replicates of the same age and infection status clustered tightly together in the principal component space, except for the salivary gland sporozoite-infectious samples (14 dpi), demonstrating that variation in the libraries due to handling and processing was successfully minimised. The separation of the four libraries of the 14 dpi samples into two clusters correlates with demonstrated differences in parasite load of the mosquitoes (Fig. 2a; Supplementary Fig. 3).

Antennal transcripts were significantly differentially regulated between the two age-matched cohorts (6187), of which 3 465 were differentially abundant only between 14 dpi and 14 dc, whereas in 7 dpi and 7 dc, 850 were differentially abundant (Fig. 2b). In total, 4807 transcripts were significantly differentially regulated between the two age-matched control groups and the two groups carrying *P. falciparum* parasites, of which 1071 transcripts were shared between them (Fig. 2c). Within the age-matched control groups, 2614 transcripts were uniquely regulated, whereas 1122 were uniquely regulated within the two groups carrying *P. falciparum* parasites (Fig. 2c; Supplementary Fig. 3). Overall, differentially abundant transcripts were not condition-dependently regulated, except those regulated post-infection during both the midgut oocyst-infected (7 dpi) and salivary gland sporozoite-infectious (14 dpi) stages (1872), of which more than 80% of the transcripts were down-regulated post-infection compared with the antennal transcripts of the uninfected, age-matched, post-blood meal females (Fig. 2b).

**Figure 3:**
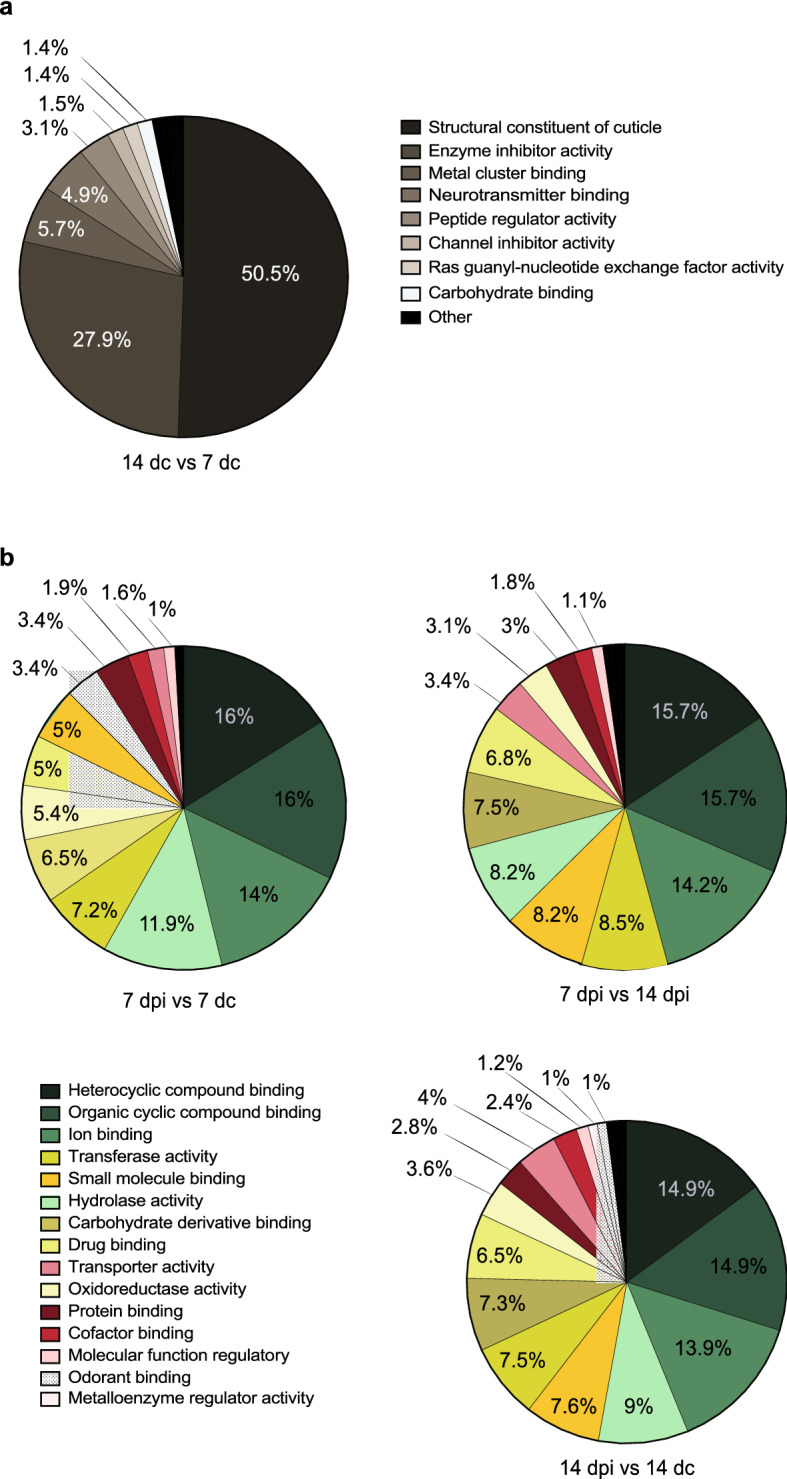
*Plasmodium falciparum* infection stage affects the functional ontology of differentially abundant antennal transcripts. A level 3 gene ontology analysis of molecular function of *Anopheles gambiae* antennal transcriptomes comparing those of 13 and 19 days post-emergence females (7 days control, dc and 14 dc, respectively) (**a**), as well as those of 7 and 14 days post-infection (dpi) females along with age-matched controls (**b**). Gene ontology classes that were included comprised greater than 1% of the total number of differentially abundant genes identified. Those with less than 1% are grouped together in “other”.

To characterise the functional ontology of the differentially abundant genes in the antennae of midgut oocyst-infected (7 dpi) and salivary gland sporozoite-infectious (14 dpi) mosquitoes with their age-matched controls, a gene ontology (GO) analysis of molecular function (level three) was conducted (Fig. 3). Of the 3 685 differentially abundant transcripts between the two uninfected control groups, the majority (> 75%) were functionally classified as structural constituent of cuticle (GO: 0042302) and enzyme inhibitor activity (GO: 0004857; Fig. 3a). Both of these classes were more abundant in older compared to the younger individuals. None of the age-dependent GO terms identified in the uninfected cohort comparison (Fig. 3a) were detected in the pairwise comparisons of the antennal transcriptomes among midgut oocyst-infected (7 dpi), salivary gland sporozoite-infectious (14 dpi) and their age-matched controls. The three most represented functional classes in the pairwise comparisons between the midgut oocyst-infected (7 dpi) and salivary gland sporozoite-infectious (14 dpi) groups with their age-matched controls, as well as between the midgut oocyst-infected (7 dpi) and salivary gland sporozoite-infectious (14 dpi) antennal transcriptomes, were heterocyclic compound binding (GO: 1901363), organic cyclic compound binding (GO: 0097159) and ion binding (GO: 0043167; Fig. 3b). Two functional classes were regulated differently in the antenna of mosquitoes with a *P. falciparum* infection, odorant binding (GO: 0005549) and carbohydrate derivative binding (GO:0097367). The number of genes in the functional class odorant binding were regulated in both midgut oocyst-infected (7 dpi) and salivary gland sporozoite-infectious (14 dpi) samples, while those in the carbohydrate derivative binding class were differentially regulated only in infectious samples (Fig. 3b).

**Figure 4:**
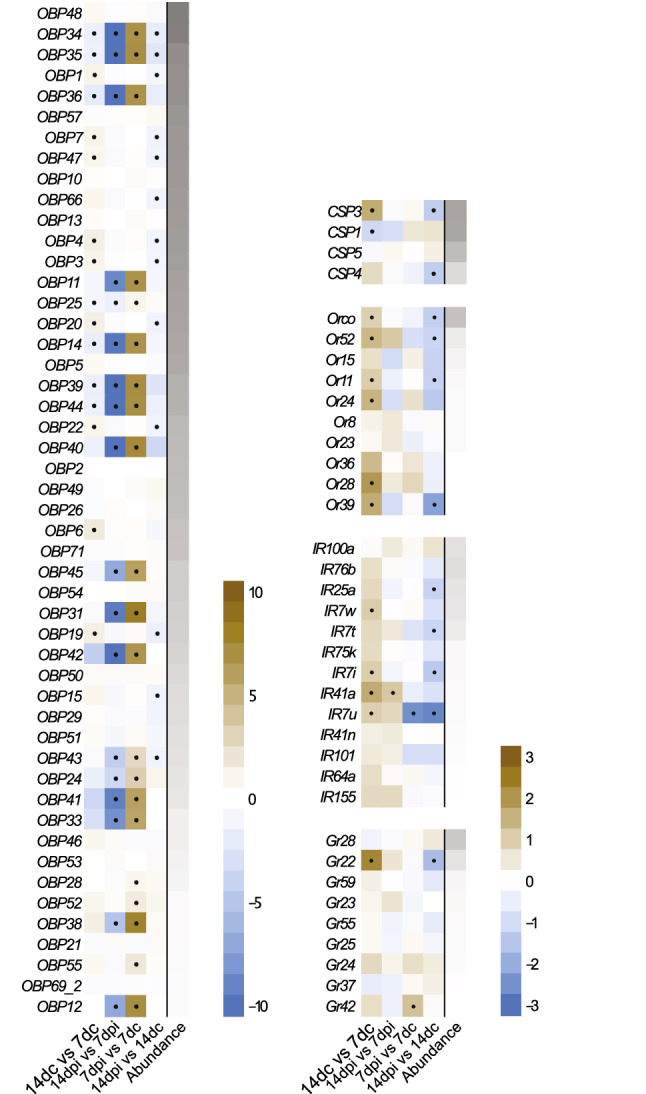
Antennal chemosensory transcript abundance in female *Anopheles gambiae* is affected by *Plasmodium falciparum* infection stage. Chemosensory transcripts demonstrating reliable expression (> 1 TPM) in the antennae of midgut oocyst-infected (7 days post-infection; dpi) and salivary gland sporozoite-infectious (14 dpi) female mosquitoes, along with their age-matched controls (7 days control; 7dc and 14 dc) were analysed in pairwise comparisons displayed as log_2_ fold change in abundance. Chemosensory genes are arranged into gene families, odorant binding proteins (*OBP*s), chemosensory proteins (*CSP*s), odorant receptors (*Or*s), ionotropic receptors (*Ir*s) and gustatory receptors (*Gr*s), in descending order of abundance (grey gradient bar, log_10_ TPM). Significant differences in abundance (fold change > 2; FDR-corrected *p* < 0.05) are indicated by black dots.

A detailed analysis of the major chemosensory gene families associated with the odorant binding functional class, *Or*s, *Ir*s, *Gr*s, *Csp*s and *Obp*s, was conducted. As the mosquitoes aged post-blood meal, 34% of the chemosensory genes were significantly regulated in the antennae of uninfected females, with chemosensory receptors demonstrating higher abundance in the older females, while the binding proteins were both up- and down-regulated (Fig. 4). Following *P. falciparum* infection, the abundance of 18 *Obps* reduced with age post-blood meal, while only one chemosensory gene, *Ir41a*, demonstrated an increased abundance in the antennae of older females (Fig. 4). The differential abundance of these 18 *Obp*s, along with three others, appear to be a result of an increased abundance at 7 dpi compared to the age-matched controls. The other chemosensory gene that was regulated at this age, *Ir7u*, was down-regulated upon infection.

The post-blood meal, age-dependent regulation of chemosensory genes was affected following *P. falciparum* infection, as 73% of those genes that were up-regulated with age (Fig. 4, column 1), were shown not to increase in abundance post-infection (Fig. 4, columns 2 and 3), resulting in a higher abundance of these transcripts in the controls compared with those at 14 dpi (Fig. 4, column 4). The only exception to this was *Ir7u*, which was down-regulated at 7 dpi, exacerbating the decreased abundance observed at 14 dpi. Interestingly, when the 7 dpi mosquitoes were compared to 14 dpi, the abundance of the above-mentioned *Obps* were reversed, returning to pre-infection levels (Fig. 4, column 2).

## Discussion

The stage of infection of *P. falciparum* in *An. gambiae* has a significant impact on vector-related behaviour, which affects malaria transmission and disease dynamics^2,5,27,28^. This study confirms that midgut oocyst-infected (7 dpi) mosquitoes are less active than salivary gland sporozoite-infectious (14 dpi) females, and furthermore identifies this change in behaviour to be a result of an interaction between infection stage and age post-blood meal, which can be modulated by the presence of human odour and diel rhythms. We demonstrate that transcriptomic profiling of antennal tissue of *P. falciparum*oocyst-infected (7 dpi) and salivary gland sporozoite-infectious (14 dpi) *An. gambiae* identifies members of several chemosensory gene families as changing with age post-blood meal and infection status, suggesting a molecular mechanism, which may regulate the sensitivity of the peripheral olfactory system of the vector to human odour during the sporogonic cycle. As age and a blood meal are intrinsic to the infection status of the female, controlling for age post-blood meal provides a more accurate indication of which behaviours and molecular correlates are regulated by *P. falciparum* infection.

Host seeking in mosquitoes is dependent on age^20,21,23,29,30^ and physiological state, including infection^3–5^. Our results demonstrated that the overall locomotor activity of uninfected females significantly reduced with age post-blood meal, in line with previous studies on mosquitoes^21,23^, further emphasising the role of age in regulating host seeking. As mosquitoes age, energy reserves decrease, resulting in older females reducing their search activities and becoming more selective to reduce their energy requirements^31,32^. We find that the presence of human odour did not alter the overall locomotor activity, similar to that described by Cator et al.^10,28^, suggesting that the difference in the level of activity with age is independent of the presence or absence of human odour. While previous studies on the effects of *Plasmodium* infection on mosquito host seeking have shown a reduced activity in midgut oocyst-infected (7 dpi) compared with salivary gland sporozoite-infectious (14 dpi) mosquitoes, these have not controlled for the age of the insects^10,11^. Our results indicate an interaction between age post-blood meal and infection in midgut oocyst-infected females (7 dpi), which display a similar locomotor activity as an age-matched cohort, is not seen in salivary gland sporozoite-infectious individuals (14 dpi). When controlling for age post-blood meal and observing the effect of infection, midgut oocyst-infected females reduced their overall locomotor activity compared to age-matched controls in the absence of human odour, while in the presence of human odour there was no discernible difference in activity. In contrast, salivary gland sporozoite-infectious (14 dpi) mosquitoes increased their overall locomotor activity in the presence of human odour, but not in its absence, compared to age-matched controls. These findings provide an explanation for the discrepancies between past studies on the behaviour of oocyst-infected (7 dpi) and sporozoite-infectious females (14 dpi)^10,33^. The mechanisms underlying the changes in host-seeking and blood-feeding behaviours are still unclear, however several hypotheses have been advanced, chief among these is that the response in midgut oocyst-infected (7 dpi) and salivary gland sporozoite-infectious females (14 dpi) is a result of a general immune response, and/or a direct manipulation of the vector behaviour by the pathogen^3,34–36^. The increased response to human odour, by salivary gland sporozoite-infectious mosquitoes (14 dpi), coincides with increased feeding frequency, probing rate and meal size^28,35,37,38^, and has substantial effects on malaria transmission ecology and overall disease dynamics^36^.

Diel locomotor activity in mosquitoes is regulated by the age post-blood meal and infection status of female malaria vectors^26,39,40^. In the presence and absence of human odour, younger uninfected mosquitoes (13 days post-emergence, dpe) were more active than the older mosquitoes (19 dpe) during photophase, scotophase and peaking at dawn^41^. We find that the peak of activity for older females, on the other hand, occurred at dusk. We note, however, that the continuous temporal analysis of the locomotor activity, in which other factors are not taken into consideration, revealed an increased activity of older females during scotophase in the absence of human odour. Post-blood meal, the age-dependent change in diel locomotor activity was not modulated by the presence of human odour, which is in line with the diel patterns of the peripheral olfactory system responsiveness^42–44^. The shift in peak activity of older females co-occurs with an increased propensity of older females to visit nectar sources at dusk^45^, indicating a behavioural compartmentalisation due to an age-related reduction in energy reserves^31,46^. Here, we show infection with *P. falciparum* altered the diel locomotor activity in both the presence and absence of human odour, generally decreasing activity in midgut oocyst-infected (7 dpi) and salivary gland sporozoite-infectious females (14 dpi) in the absence of human odour, similar to that described in *An. stephensi* infected with *Plasmodium yoelii*^47^. In the presence of human odour, midgut oocyst-infected (7 dpi) and salivary gland sporozoite-infectious females (14 dpi) demonstrated a disrupted activity pattern associated with host seeking. In particular, infectious females (14 dpi) shifted their odour-associated activity to scotophase and dawn, which is in line with observations of field-caught mosquitoes carrying sporozoites^39,48^. By selectively increasing their activity during times of low-risk of predation and high access to human hosts, the infectious females increase the probability of a successful blood meal and pathogen transmission^49–53^. While the infectivity of the bite is not affected by diel rhythms^54^, the altered host-related activity pattern in infectious females may have explicit implications on the most effective deployment of vector control tools, and thus our ability to affect malaria transmission dynamics efficiently^36^. Whether this is a result of an active manipulation by the pathogen or a general immune response remains to be clarified^3,34–36,55,56^.

Our results demonstrate age post blood-meal and *P. falciparum* infection significantly affected overall antennal transcript abundance, with infection systemically down-regulating gene abundance, and differentially affecting the functional ontological class odorant binding (GO:0005549). Among the differentially abundant transcripts, antennal abundance generally increased with age across all of the major olfactory-related gene families, whereas only *Obp*s, the most abundantly expressed transcripts of the chemosensory gene families, exhibited higher abundance in midgut oocysts-infected (7 dpi) compared with salivary gland sporozoites-infectious females (14 dpi). Odorant binding proteins are involved in binding and transporting odorants to the receptors and in the overall sensitivity of the peripheral olfactory system by providing gain control^22,57,58^. The Obps appear to play a major role in regulating the sensitivity and selectivity of the peripheral olfactory system of *An. gambiae* in response to ageing and infection status, as 67% of the *Obp* transcripts reliably expressed in the antennae were age- and infection-dependently regulated. As the females age, older individuals exhibited a higher transcript abundance in two thirds of the differentially expressed classic, and all of the plus-C classified *Obps*^17,57^. The other third of the differentially regulated classic *Obps*, along with all 13 of the two-domain *Obps*^17,57^, were expressed abundantly in 7 dpi antennae, compared with not being reliably expressed in their age-matched controls and salivary gland sporozoite-infectious females (14 dpi). Bimolecular protein interaction models indicate that the most energetically favourable ligands of the reliably expressed classic *Obps*, including those that were differentially abundant, are distinct sets of carboxylic acids, aromatics and terpenes^17^, known to regulate host and floral seeking^45,46^. While no clear pattern of ligand binding was apparent associated with ageing, those classic *Obps* increasing in abundance 7 dpi generally did not include carboxylic acids among their modelled ligands, likely resulting in a reduced sensitivity of the peripheral olfactory system to these salient human odorants by midgut oocyst-infected females (7 dpi)^59–61^. This corresponds to the reduction in human odour-associated activity exhibited by midgut oocyst-infected females (7 dpi).

The functionally characterised differentially abundant olfactory receptors, *Or11*, *Or39* and *Ir41a*, all respond to human odour constituents^16,21,62^. The receptor *Or39* has been postulated to modulate the onset of host seeking in *Anopheles coluzzii* through the concerted downregulation of the receptor and the sensitivity to sulcatone of its cognate olfactory sensory neuron^21^. Whether the lower abundance of *Or39*, in conjunction with *Or11*, observed in the antennae of salivary gland sporozoite-infectious (14 dpi) compared with uninfected females, underlies the observed increased locomotor activity of salivary gland sporozoite-infectious females (14 dpi) in the presence of human odour, remains to be investigated. The only receptor to have a higher abundance in the antennae of salivary gland sporozoite-infectious (14 dpi) compared to midgut oocyst-infected females (7 dpi), *Ir41a*, responds to the human spermous cyclic amine, pyrrolidine^16,63^, suggesting that this receptor is involved in the increased attraction to human odour 14 dpi. The differential regulation of transcript abundance of genes modulating the chemosensory pathway in response to parasite development within the insect vector provides a mechanism by which the physiological^14^ and behavioural sensitivity^4,10,11,33,35^ to human odour can be modulated during the sporogonic cycle of *P. falciparum*.

Behavioural activity of *An. gambiae* and its expression of antennal chemosensory-related genes are altered in an age- and *P. falciparum* stage-dependent manner. Irrespective of the mechanism regulating these changes, be it active manipulation by the parasite and/or a general response to immune challenge, the differential behavioural response to human odour of females as they age or become infected post-blood meal significantly affects vectorial capacity and malaria transmission. Our ability to increase the resolution concerning which behavioural change is affected by age and/or infection, allows us to fine tune the epidemiological models for malaria transmission^33^, and integrate both factors and their interactions into the development and implementation of control measures.

## Methods

### Ethics

Human blood (type O) was provided in citrate–phosphate–dextrose–adenine anti-coagulant/preservative, and serum (type AB) was obtained from the blood transfusion service (blood bank) at the Karolinska University Hospital (Solna, Sweden), in accordance with the Declaration of Helsinki, and approved by the Ethical Review Board in Stockholm (2011/850-32). The human materials were obtained anonymously from the blood bank and did not require an informed consent statement. The Swedish Work Environment Authority (Stockholm, Sweden, SU FV-2.10.2-2905-13/31-01-2022) approved the class 3 biological agent laboratory and its practices, including insectary design and equipment to work with *P. falciparum*-infected mosquitoes. The regulations are mainly based on the EU directive 2000/54/EC on the protection of workers from risks related to exposure to biological agents at work.

### Mosquito rearing and blood feeding

*Anopheles gambiae* (Keele) were reared and maintained at 27 ± 1 °C, 80 ± 1% relative humidity and a photoperiod of 12 h light: 12 h dark cycle, 11 h full light of ~ 300 lx and 11 h darkness, separated by 1 h dawn and dusk transitions, respectively. The Keele colony was established at Keele University as a result of balanced interbreeding of four *An. gambiae s.l*. strains: G3 from MacCarthy Island, the Gambia; and three Tanzanian strains, ZAN U from Zanzibar, Ifakara strain from Njage, and KIL from Marangu^64^, and set up at Stockholm University in 2014. For experiments, pupae (ca. 350) were collected and transferred to cages (custom made: 30 cm × 20 cm × 20 cm) to emerge as adults, which were fed ad libitum on 5% glucose and 0.05% para-amino benzoic acid (PABA, Sigma-Aldrich, Steinheim, Germany). Females (5 dpe) were provided with a meal of cultured red blood cells (RBCs) through glass membrane-feeders connected to a custom-built heating system kept at 37 °C. The RBCs were washed with Roswell Park Memorial Institute (RPMI-1640) medium (Gibco, ThermoFisher Scientific, Drieich, Germany), and stored in RPMI-1640 at 50% haematocrit at 4 °C prior to use.

### Parasite culture and mosquito infection

The strain of *P. falciparum* used in this study, NF_54_-SU, was initially isolated in the Netherlands and donated by Klavs Berzins. In vivo culturing of *P. falciparum* NF_54_-SU followed the standard protocol^65^. In short, *P. falciparum* NF_54_-SU was cultured using washed RBCs (5% haematocrit) from type-O blood donors, and 10% serum mixed with RPMI-1640 medium (Gibco™, ThermoFisher Scientific), supplemented with 5.94 gl^−1^ HEPES buffer (Sigma Aldrich), 2.1 g l^−1^ sodium hydrogen carbonate (Sigma Aldrich), and 0.05 g l^−1^ hypoxanthine (Sigma Aldrich). The cultures were kept in controlled conditions under gas (1% O_2_, 3% CO_2_ and 96% N_2_) at 37 °C. *Plasmodium falciparum* gametocyte cultures were set up at 0.5–0.7% parasitaemia, 6% haematocrit in RPMI-1640 medium. On the day of experimental infection, uninfected RBCs were supplemented with *P. falciparum* gametocytes to a final gametocytaemia of ca. 0.7%, which generates a high infection prevalence (> 50%) in *An. gambiae s.s.*^65^. For each infectious meal, mixtures of gametocytes from 14- and 17-day gametocyte cultures were used. Twenty-eight groups of ca. 100 mosquitoes were fed on mature gametocyte-infected RBCs or on uninfected washed RBCs using a membrane feeder. All age-matched control females (7- and 14-day control; 7 and 14 dc) were handled in the same manner as the infected and infectious cohorts, including access to mates, blood meals and oviposition substrates.

### Mosquito odour-mediated locomotor activity

For recording the behavioural activity of the mosquitoes, DAM2 *Drosophila* activity monitors (TriKinetics Inc., Waltham, MA, USA) were used (Fig. 1a; Supplementary Fig. 4). Two hours prior to the experiments, mosquitoes were placed individually in glass tubes (65 mm × 7 mm), using an aspirator, for pre-conditioning. Cotton plugs saturated with 5% glucose with 0.05% PABA (as above) were placed at one end of the glass tubes to provide ad libitum access to sustenance. Human odour was supplied by a previously worn cotton sock (ca. 24 h) placed inside a 200 ml glass wash bottle (Sigma Aldrich). The odour was introduced, via Teflon tubing, into the glass tubes through a MAN2 gas distribution manifold (TriKinetics Inc.). A clean unworn sock was similarly used as a control. Each experiment was replicated three times with midgut oocyst-infected (7 dpi) and salivary gland sporozoite-infectious (14 dpi) mosquitoes assessed in parallel with age-matched controls, which were blood fed at the same time as those which were infected, for a total of 384 mosquitoes assayed (96 per condition). Behavioural experiments were conducted in the class 3 biological agent laboratory (BSL3) within a climate chamber kept at 27 ± 1 °C, 70% relative humidity with a photoperiod of 11 h light: 11 h dark with 60 min of both dawn and dusk lighting conditions. Both assays were conducted over 24 h, with activity data recorded every 30 s. In this assay, the activity of female *Anopheles gambiae*, 7 days post-infection (dpi) and 14 dpi with *P. falciparum*, compared to age-matched uninfected females (days control, dc), in the presence (with human odour) or absence of human odour (w/o human odour) were assessed. Individual locomotor profiles (n = 384), assessed for variation using a redundancy analysis, demonstrated a differential effect of age post-blood meal (Fig. 1a; Supplementary Fig. 4a,b).

The influence of age, the presence and absence of human odour, and the effect of the sporogonic stages of *P. falciparum* on the *An. gambiae* (7 and 14 days post infection; 7 and 14 dpi) diel locomotor activity was analysed using general linear mixed models (GLMM), in which the effect of replication (experimental blocks) and the weighting of multiple replications (random variables) were taken into account. For all results, the significance of the best maximal model containing all explanatory effects was evaluated by using likelihood ratio test. All analyses were performed using R (R Core team^66^: R v.3.2.3 and RStudio 1.1.463^67^). The *β*-estimated values of locomotor activity were estimated in all final significant models (GLMM: lmer function; in Fig. 1b: The mixed model was build based on the effect of age as a main fixed variable combined with the random effect of the other variables; Fig. 1c: The model demonstrated the effect of the main fixed variable of infection, including the rest of the variables as random variables; Fig. 1d: The mixed model estimated the effect of all four main fixed variables including age, infection, time, human odour, as well as the random effect of the experimental replication combined with the interactions among all these variables).

To assess the average overall locomotor activity of all the mosquitoes across the 24 h (Supplementary Fig. 1a–d) of recording, the Williams mean was used. This geometric mean (log (n + 1)) was calculated for all recordings every 30 min, and minimizes the influence of low/high values and inflated numbers of zeros on the data distribution. To compare the Williams means, a repeated measures two-way ANOVA with a Tukey’s post hoc analysis was used to compare the overall locomotion activity every 30 min at 7 dpi and 14 dpi. The significance of the test is given by the Williams mean, which is simple moving average, separately for only one fixed explanatory variable at a time. This method does not allow for, or include the variation from, an extra factor, such as experimental replication (random variable in the case of biological and ecological studies). In contrast, GLMM statistical modelling (the output of a mixed model) takes into account the whole data set by including the effect of replication (i.e., experimental blocks, infection, age, diel activity pattern and host odour). Therefore, GLMM: mixed model (lmer) is an appropriate method for analysing the complex biological and ecological studies when the response variable, in this case locomotion, is affected by multiple factor interactions as it accounts for multiple replicates and explanatory variable interactions. In all analyses, all of the data met the assumptions of the test for normality and error homogeneity. Backward elimination was used for sequential removal of non-significant variables, to obtain the minimal statistically significant model. All graphical visualisations and statistical analyses were preformed using GraphPad Prism^68^ and R (R Core team^66^: R v.3.2.3 and RStudio 1.1.463^67^). The precise levels of significance are presented in the results section, except the universally accepted *p* < 0.001.

### Transcriptome analyses

Pairs of antennae from midgut oocyst-infected (7 dpi), salivary gland sporozoite-infectious (14 dpi) and age-matched control mosquitoes, all of which received a blood meal at the same age, were collected from individual females following flash freezing on dry ice for < 30 s, and placed into 24-well culture plates (ThermoFisher Scientific) in RNAlater (500 µl; Thermo Fisher Scientific). Individual carcasses (bodies missing antennae) of midgut oocyst-infected (7 dpi) and salivary gland sporozoite-infectious (14 dpi) mosquitoes were also collected for subsequent qPCR analysis, to determine the parasite abundance of individual females (see below). Following the quantification of the parasite load, the antennae of successfully infected individuals were pooled into tubes of 150 pairs of antennae, according to the level of infection, and stored at − 80 °C. Four replicates of each condition were collected. RNA libraries were constructed using a TruSeq RNA Library Prep Kit (Illumina, Berlin, Germany) from total RNA from the pooled antennae following extraction and DNase I digestion (RNeasy Mini kit, Qiagen, Hilden, Germany). Quality and quantity control of total RNA aliquots was performed using a Nanodrop spectrophotometer (Thermo Fisher Scientific), an Agilent 2100 Bio-analyser (Santa Clara, CA, USA) and a Qubit 2.0 Fluorometer (Thermo Fisher Scientific), before sending to BGI Genomics (MGI Tech Co., Ltd., China) for Illumina paired-end indexed sequencing, using the Illumina HiSeq2000.

### RNA-Seq and differential expression analyses

High-quality reads were determined using the following criteria: low quality reads were clipped from the start and end of each read using a sliding window and reads shorter than 40 nt were removed. The cleaned reads were mapped using CLC Genomics Workbench version 11 (https://digitalinsights.qiagen.com) to the *An. gambiae* genome Agam4 with reference to the Agam4.10 gene set (www.vectorbase.org). On average 94.11% of all reads mapped to the genome. Transcript abundance was reported as transcript per million (TPM) with a threshold level of abundance above 1 TPM. Differential transcript abundance was determined using the *β*-binomial general linear model algorithms in CLC Genomics Workbench 11. To control for false discovery rate (FDR), the Benjamini–Hochberg correction was applied^69^. This analysis generated weighted fold changes (FC) and FDR-corrected *p*-values that were used to detect differential expression. Significantly differential gene abundance was determined based on a FC ≥ 2 and an FDR-corrected *p*-value < 0.05.

### Quantitative real-time polymerase chain reaction analysis

Quantitative real-time polymerase chain reaction (qPCR) was used to estimate the abundance of *P. falciparum* in individual mosquitoes at 7 and 14 days after being subjected to an infectious meal, and used for both behavioural and transcriptomic analyses, allowing quantification of the numbers of parasite genomes. In all analyses, a single body of an uninfected female mosquito was used as a negative control. Reactions were performed on a Roche Light Cycler using SYBR Green (Roche, Mannheim, Germany). DNA standards containing known numbers of *P. falciparum* parasites were produced from asexual cultures, and used to generate standard curves of *P. falciparum*. A similar statistical approach, as described for the behavioural analyses, was used to test for variation in the number of parasites (oocyst load and total number of sporozoites per whole mosquito body)^70^. Given the highly over-dispersed nature of parasite abundance data, a negative binomial distribution was assumed in these analyses (GLMM: glmmADMB, nlme package; R Core team^66^: R v.3.2.3 and RStudio 1.1.463^66^). A backwards elimination approach was used to test for the significance of all fixed effects and interactions, while controlling for random variation due to replicate, as for the analyses of oocyst and sporozoite prevalence.

## Supplementary Information

Supplementary Information.

## Data Availability

All data supporting the findings of this study are available within the article and its Supplementary Information files. The sequencing data are available at the NCBI database (BioProject ID PRJNA756244), and are available from the corresponding authors upon request.

## References

[1] De Moraes CM (2014). Malaria-induced changes in host odors enhance mosquito attraction. Proc. Natl. Acad. Sci. USA.

[2] Emami SN (2017). A key malaria metabolite modulates vector blood seeking, feeding, and susceptibility to infection. Science.

[3] Hurd H (2003). Manipulation of medically important insect vectors by their parasites. Annu. Rev. Entomol..

[4] Lefevre T, Thomas F (2008). Behind the scene, something else is pulling the strings: Emphasizing parasitic manipulation in vector-borne diseases. Infect. Genet. Evol..

[5] Tallon AK (2020). Dengue infection modulates locomotion and host seeking in *Aedes aegypti*. PLoS Negl. Trop. Dis..

[6] Botto-Mahan C, Cattan PE, Medel R (2006). Chagas disease parasite induces behavioural changes in the kissing bug *Mepraia spinolai*. Acta Trop..

[7] Lefevre T (2007). Malaria *Plasmodium* agent induces alteration in the head proteome of their *Anopheles* mosquito host. Proteomics.

[8] Biron DG (2005). Behavioural manipulation in a grasshopper harbouring hairworm: A proteomics approach. Proc. R. Soc. B.

[9] Beier JC (1998). Malaria parasite development in mosquitoes. Annu. Rev. Entomol..

[10] Cator LJ (2013). 'Manipulation' without the parasite: Altered feeding behaviour of mosquitoes is not dependent on infection with malaria parasites. Proc. Biol. Sci..

[11] Smallegange RC (2013). Malaria infected mosquitoes express enhanced attraction to human odor. PLoS ONE.

[12] Cornet S, Nicot A, Rivero A, Gandon S (2019). Avian malaria alters the dynamics of blood feeding in *Culex pipiens* mosquitoes. Malar. J..

[13] Padilha KP (2018). Zika infection decreases *Aedes aegypti* locomotor activity but does not influence egg production or viability. Mem. Inst. Oswaldo Cruz.

[14] Stanczyk NM (2019). Species-specific alterations in *Anopheles* mosquito olfactory responses caused by *Plasmodium* infection. Sci. Rep..

[15] Lu T (2007). Odor coding in the maxillary palp of the malaria vector mosquito *Anopheles gambiae*. Curr. Biol..

[16] Pitts RJ, Derryberry SL, Zhang Z, Zwiebel LJ (2017). Variant ionotropic receptors in the malaria vector mosquito *Anopheles gambiae* tuned to amines and carboxylic acids. Sci. Rep..

[17] Manoharan M (2013). Comparative genomics of odorant binding proteins in *Anopheles gambiae*, *Aedes aegypti*, and *Culex quinquefasciatus*. Genome Biol. Evol..

[18] Bohbot JD (2011). Conservation of indole responsive odorant receptors in mosquitoes reveals an ancient olfactory trait. Chem. Senses.

[19] Erdelyan CN, Mahood TH, Bader TS, Whyard S (2012). Functional validation of the carbon dioxide receptor genes in *Aedes aegypti* mosquitoes using RNA interference. Insect Mol. Biol..

[20] Omondi BA, Majeed S, Ignell R (2015). Functional development of carbon dioxide detection in the maxillary palp of *Anopheles gambiae*. J. Exp. Biol..

[21] Omondi AB, Ghaninia M, Dawit M, Svensson T, Ignell R (2019). Age-dependent regulation of host seeking in *Anopheles coluzzii*. Sci Rep.

[22] Rinker DC (2013). Blood meal-induced changes to antennal transcriptome profiles reveal shifts in odor sensitivities in *Anopheles gambiae*. Proc. Natl. Acad. Sci. USA.

[23] Tallon AK, Hill SR, Ignell R (2019). Sex and age modulate antennal chemosensory-related genes linked to the onset of host seeking in the yellow-fever mosquito, *Aedes aegypti*. Sci. Rep..

[24] Hill SR, Ignell R (2021). Modulation of odour-guided behaviour in mosquitoes. Cell Tissue Res..

[25] Hill SR, Ghaninia M, Ignell R (2019). Blood meal induced regulation of gene expression in the maxillary palps, a chemosensory organ of the mosquito *Aedes aegypti*. Front. Ecol. Evol..

[26] Jones MDR, Gubbins SJ (1978). Changes in the circadian flight activity of the mosquito *Anopheles gambiae* in relation to insemination, feeding and oviposition. Physiol. Entomol..

[27] Koella JC, Sorensen FL, Anderson RA (1998). The malaria parasite, *Plasmodium falciparum*, increases the frequency of multiple feeding of its mosquito vector, *Anopheles gambiae*. Proc. Biol. Sci..

[28] Anderson RA, Koella JC, Hurd H (1999). The effect of *Plasmodium yoelii* nigeriensis infection on the feeding persistence of *Anopheles stephensi* Liston throughout the sporogonic cycle. Proc. R. Soc. B.

[29] Bohbot JD, Durand NF, Vinyard BT, Dickens JC (2013). Functional development of the octenol response in *Aedes aegypti*. Front. Physiol..

[30] Foster WA, Takken W (2004). Nectar-related vs. human-related volatiles: behavioural response and choice by female and male *Anopheles gambiae* (Diptera: Culicidae) between emergence and first feeding. Bull. Entomol. Res..

[31] Rowley WA, Graham CL (1968). The effect of age on the flight performance of female *Aedes aegypti* mosquitos. J. Insect. Physiol..

[32] Nayar JK, Sauerman DM (1973). A comparative study of flight performance and fuel utilization as a function of age in females of Florida mosquitoes. J. Insect. Physiol..

[33] Vantaux A (2015). Host-seeking behaviors of mosquitoes experimentally infected with sympatric field isolates of the human malaria parasite *Plasmodium falciparum*: No evidence for host manipulation. Front. Ecol. Evol..

[34] Cator LJ (2015). Immune response and insulin signalling alter mosquito feeding behaviour to enhance malaria transmission potential. Sci. Rep..

[35] Koella JC, Rieu L, Paul REL (2002). Stage-specific manipulation of a mosquito's host-seeking behavior by the malaria parasite *Plasmodium gallinaceum*. Behav. Ecol..

[36] Cator LJ, Lynch PA, Read AF, Thomas MB (2012). Do malaria parasites manipulate mosquitoes?. Trends Parasitol..

[37] Rossignol PA, Ribeiro JM, Spielman A (1984). Increased intradermal probing time in sporozoite-infected mosquitoes. Am. J. Trop. Med. Hyg..

[38] Rossignol PA, Ribeiro JM, Spielman A (1986). Increased biting rate and reduced fertility in sporozoite-infected mosquitoes. Am. J. Trop. Med. Hyg..

[39] Gillies MT (1957). Age-groups and the biting cycle in *Anopheles gambiae* A preliminary investigation. Bull. Entomol. Res..

[40] Bockarie MJ, Dagoro H (2006). Are insecticide-treated bednets more protective against *Plasmodium falciparum* than *Plasmodium vivax*-infected mosquitoes?. Malar. J..

[41] Lindsay SW, Shenton FC, Snow RW, Greenwood BM (1989). Responses of *Anopheles gambiae* complex mosquitoes to the use of untreated bednets in The Gambia. Med. Vet. Entomol..

[42] Rund SS (2013). Daily rhythms in antennal protein and olfactory sensitivity in the malaria mosquito *Anopheles gambiae*. Sci. Rep..

[43] Eilerts DF, VanderGiessen M, Bose EA, Broxton K, Vinauger C (2018). Odor-specific daily rhythms in the olfactory sensitivity and behavior of *Aedes aegypti* Mosquitoes. Insects.

[44] Tanoue S, Krishnan P, Krishnan B, Dryer SE, Hardin PE (2004). Circadian clocks in antennal neurons are necessary and sufficient for olfaction rhythms in *Drosophila*. Curr. Biol..

[45] Gary RE, Foster WA (2006). Diel timing and frequency of sugar feeding in the mosquito *Anopheles gambiae*, depending on sex, gonotrophic state and resource availability. Med. Vet. Entomol..

[46] Foster WA (1995). Mosquito sugar feeding and reproductive energetics. Annu. Rev. Entomol..

[47] Rowland M (1989). Changes in the circadian flight activity of the mosquito *Anopheles-Stephensi* Associated with Insemination, blood-feeding, oviposition and nocturnal Light-Intensity. Physiol. Entomol..

[48] Djenontin A, Bouraima A, Soares C, Egbinola S, Cottrell G (2021). Human biting rhythm of *Anopheles gambiae* Giles, 1902 (Diptera: *Culicidae*) and sleeping behaviour of pregnant women in a lagoon area in Southern Benin. BMC Res. Notes.

[49] O'Donnell AJ, Rund SSC, Reece SE (2019). Time-of-day of blood-feeding: Effects on mosquito life history and malaria transmission. Parasit. Vectors.

[50] Takken W, van Loon JJ, Adam W (2001). Inhibition of host-seeking response and olfactory responsiveness in *Anopheles gambiae* following blood feeding. J. Insect. Physiol..

[51] Reece SE, Prior KF, Mideo N (2017). The life and times of parasites: Rhythms in strategies for within-host survival and between-host transmission. J. Biol. Rhythms.

[52] Martinez-Bakker M, Helm B (2015). The influence of biological rhythms on host-parasite interactions. Trends Ecol. Evol..

[53] Mideo N, Reece SE, Smith AL, Metcalf CJ (2013). The Cinderella syndrome: Why do malaria-infected cells burst at midnight?. Trends Parasitol..

[54] Githeko AK (1993). Confirmation that *Plasmodium falciparum* has aperiodic infectivity to *Anopheles gambiae*. Med. Vet. Entomol..

[55] Poulin R (1994). The evolution of parasite manipulation of host behavior: A theoretical-analysis. Parasitology.

[56] Emami SN, Hajkazemian M, Mozuraitis R (2019). Can *Plasmodium*'s tricks for enhancing its transmission be turned against the parasite? New hopes for vector control. Pathog. Glob. Health.

[57] Li ZX, Pickett JA, Field LM, Zhou JJ (2005). Identification and expression of odorant-binding proteins of the malaria-carrying mosquitoes *Anopheles gambiae* and *Anopheles arabiensis*. Arch. Insect. Biochem. Physiol..

[58] Larter NK, Sun JS, Carlson JR (2016). Organization and function of *Drosophila* odorant binding proteins. Elife.

[59] Acree F, Turner RB, Gouck HK, Beroza M, Smith N (1968). L-Lactic acid: A mosquito attractant isolated from humans. Science.

[60] Raji JI (2019). *Aedes aegypti* mosquitoes detect acidic volatiles found in human odor using the IR8a Pathway. Curr. Biol..

[61] Cork A, Park KC (1996). Identification of electrophysiologically-active compounds for the malaria mosquito, *Anopheles gambiae,* in human sweat extracts. Med. Vet. Entomol..

[62] Carey AF, Wang G, Su CY, Zwiebel LJ, Carlson JR (2010). Odorant reception in the malaria mosquito *Anopheles gambiae*. Nature.

[63] Amoore JE, Forrester LJ, Buttery RG (1975). Specific anosmia to 1-pyrroline: The spermous primary odor. J. Chem. Ecol..

[64] Hurd H, Taylor PJ, Adams D, Underhill A, Eggleston P (2005). Evaluating the costs of mosquito resistance to malaria parasites. Evolution.

[65] Carter R, Ranford-Cartwright L, Alano P (1993). The culture and preparation of gametocytes of *Plasmodium falciparum* for immunochemical, molecular, and mosquito infectivity studies. Methods Mol. Biol..

[66] R Core Team. *R: A Language and Environment for Statistical Computing*. https://www.R-project.org/ (2020).

[67] RStudio-Team. *RStudio: Integrated Development for R*. *RStudio*. http://www.rstudio.com/ (2021).

[68] GraphPad Prism. *GraphPad Software Version 6.04*. http://www.graphpad.com/ (2020).

[69] Trapnell C (2013). Differential analysis of gene regulation at transcript resolution with RNA-seq. Nat. Biotechnol..

[70] Emami SN, Ranford-Cartwright LC, Ferguson HM (2017). The transmission potential of malaria-infected mosquitoes (*An. gambiae*-Keele, *An. arabiensis*-Ifakara) is altered by the vertebrate blood type they consume during parasite development. Sci. Rep..

