# Supplementary material for: Mosquito host-seeking diel rhythm and chemosensory gene expression is affected by age and Plasmodium stages
Source: Sci Rep. 2022 Nov 5;12:18814. doi: 10.1038/s41598-022-23529-7 (PMC9637142; doi:10.1038/s41598-022-23529-7)
Supplement: Supplementary file 1 — Supplementary Information. [file 41598_2022_23529_MOESM1_ESM.pdf]

Supplementary information

Mosquito host-seeking diel rhythm and chemosensory gene expression is affected by age and *Plasmodium* stages

Authors

Melika Hajkazemian[1†], Sharon R. Hill[2†], Raimondas Mozūraitis[3,4], Lisa Ranford-Cartwright[1,5], S. Noushin Emami[1,6,7*] & Rickard Ignell[2*]

[1]Department of Molecular Biosciences, Wenner-Gren Institute, Stockholm University, Stockholm, Sweden

[2]Disease Vector Group, Department of Plant Protection Biology, SLU, Alnarp, Sweden

[3]Laboratory of Chemical and Behavioural Ecology, Institute of Ecology, Nature Research Centre, Vilnius, Lithuania

[4]Department of Zoology, Stockholm University, Stockholm, Sweden

[5]Institute of Biodiversity, Animal Health and Comparative Medicine, College of Medical, Veterinary and Life Science, University of Glasgow, Glasgow, UK

[6]Molecular Attraction AB, Elektravägen 10, 126 30 Hägersten, Stockholm, Sweden

[7]Natural Resources Institute, FES, University of Greenwich, London, UK

This PDF file includes:

Figures: Supplementary Fig. 1 to 4

Tables: Supplementary Table 1 to 2

a)

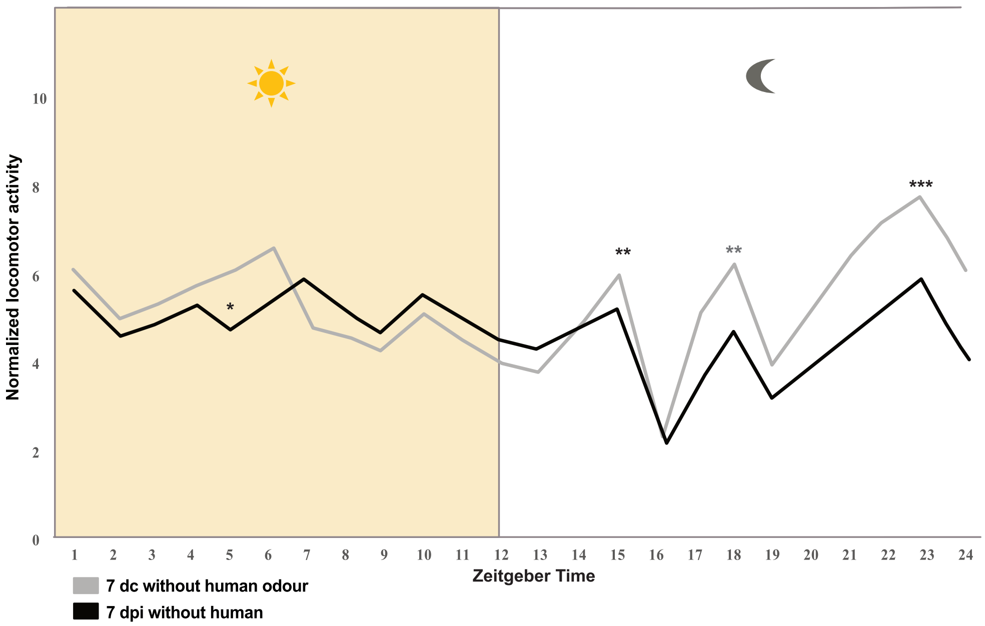

Normalized locomotor activity
0
2
4
6
8
10
*
**
**
***
1 2 3 4 5 6 7 8 9 10 11 12 13 14 15 16 17 18 19 20 21 22 23 24
Zeitgeber Time
7 dc without human odour
7 dpi without human

b)

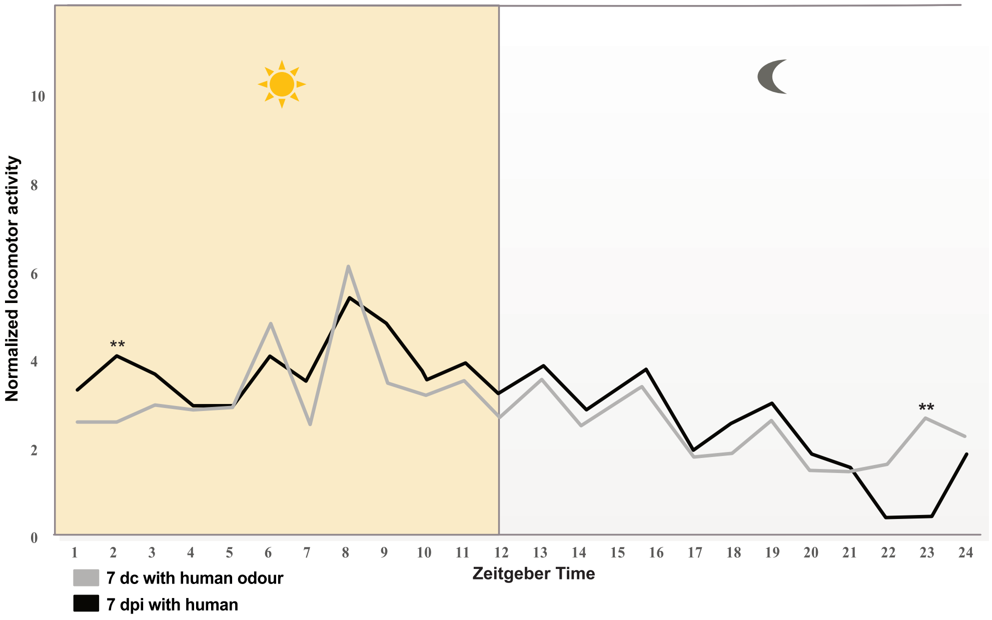

Normalized locomotor activity
0
2
4
6
8
10
**
**
1 2 3 4 5 6 7 8 9 10 11 12 13 14 15 16 17 18 19 20 21 22 23 24
Zeitgeber Time
7 dc with human odour
7 dpi with human

c)

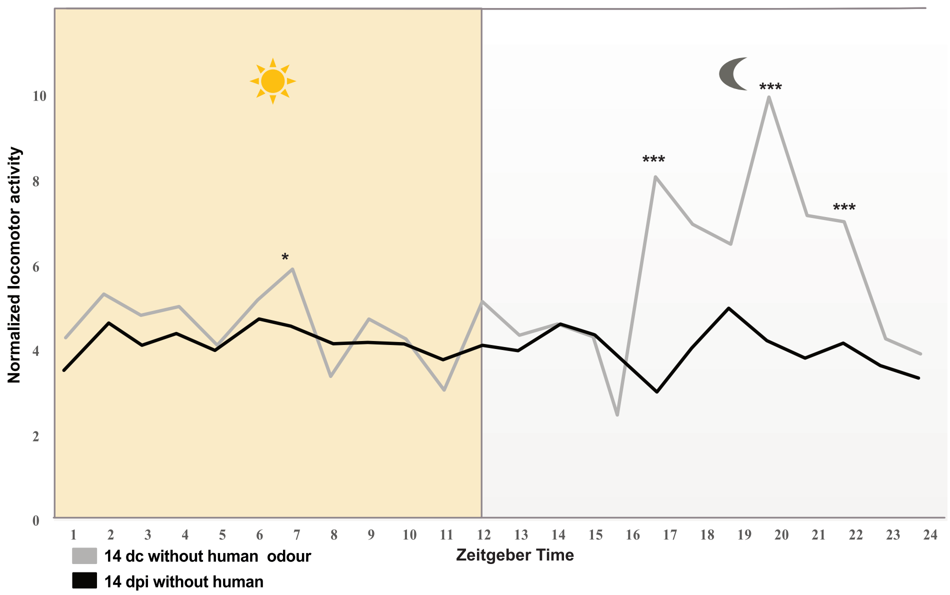

d)

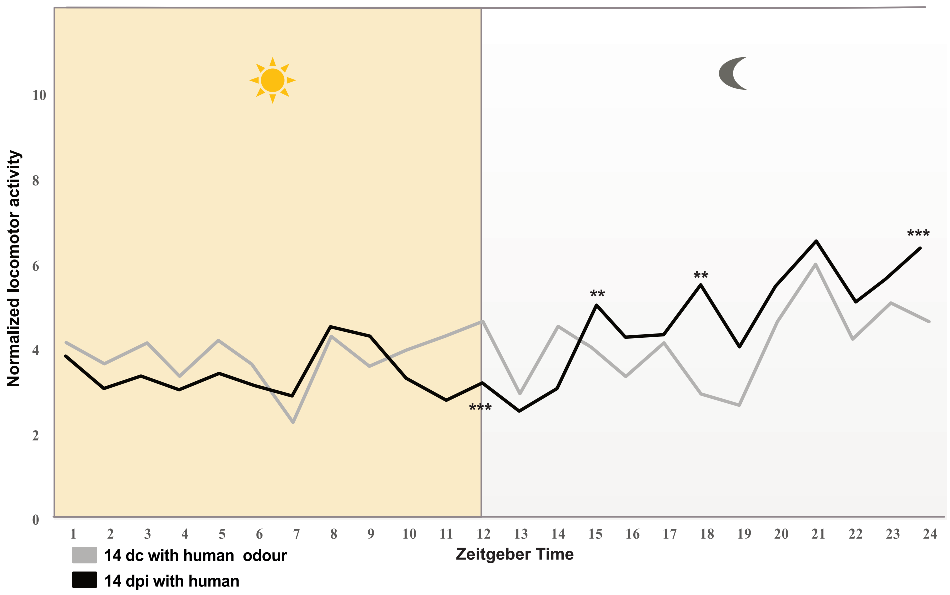

Supplementary Fig. 1. Age post-blood meal and *Plasmodium* infection differentially impacts locomotor activity. Average diel locomotor activity profiles of non-infected (grey, dc) and either 7 (a-b) or 14 (c-d) days post infection (black, dpi) female *An. gambiae*, calculated using the Williams mean, a simple moving average (SMA). A continuous temporal activity analysis provides a sliding moving average of a single factor, *i.e.,* locomotion. The yellow/grey boxes represent the 12 h scotophase. Significances were determined using a repeated measure ANOVA followed by a pairwise comparison via Tukey's post hoc test including the experimental replication. Asterisks indicate level of significance (* p < 0.05; ** p < 0.01; *** p < 0.001).

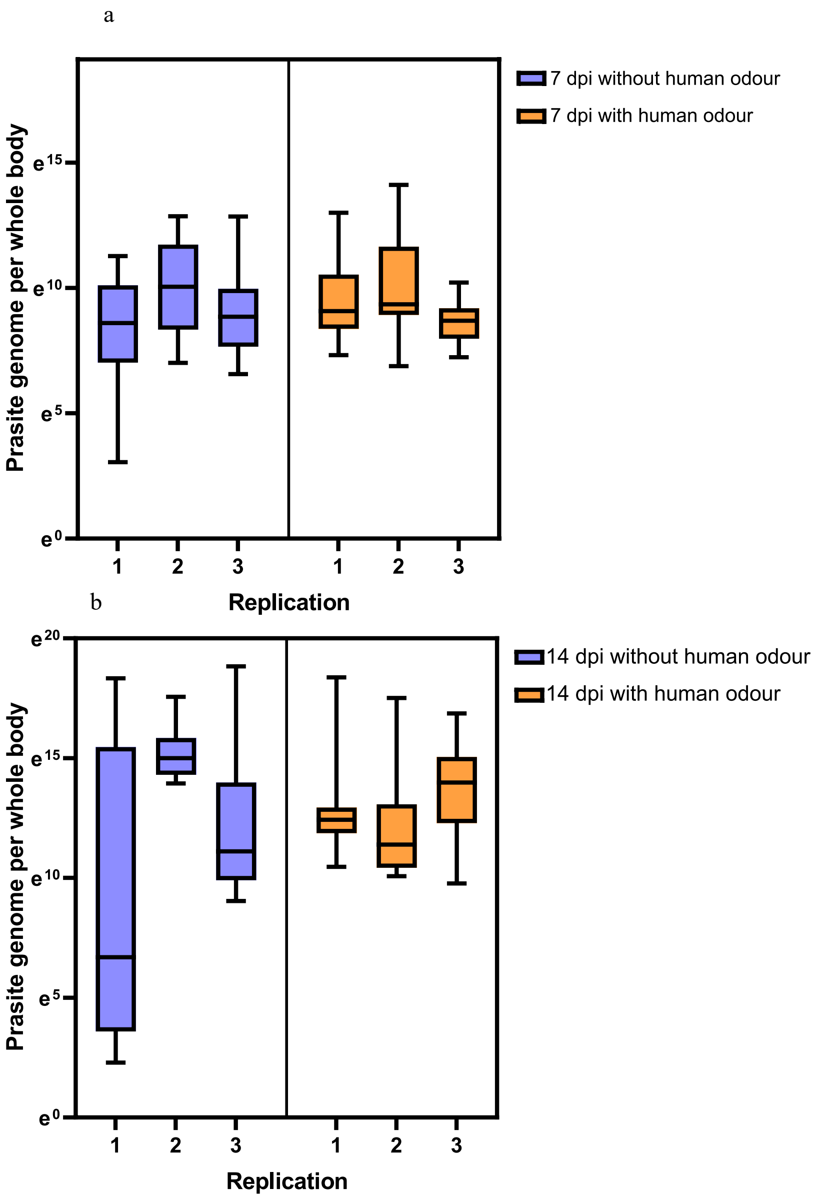

Supplementary Fig. 2.

Quantification of *Plasmodium falciparum* infection load in *Anopheles gambiae* at 7 and 14 days post-infection (dpi) for oocysts and sporozoites, respectively, with and without exposure to human odour in locomotor assays (see Figure 1). The total number of parasites per whole body of individual mosquitoes was estimated using quantitative PCR. The infection load values are taken from negative binomial model estimations. The median is represented as a thick line, the box represents the upper and lower quartile range, and the whiskers show the 95% CI.

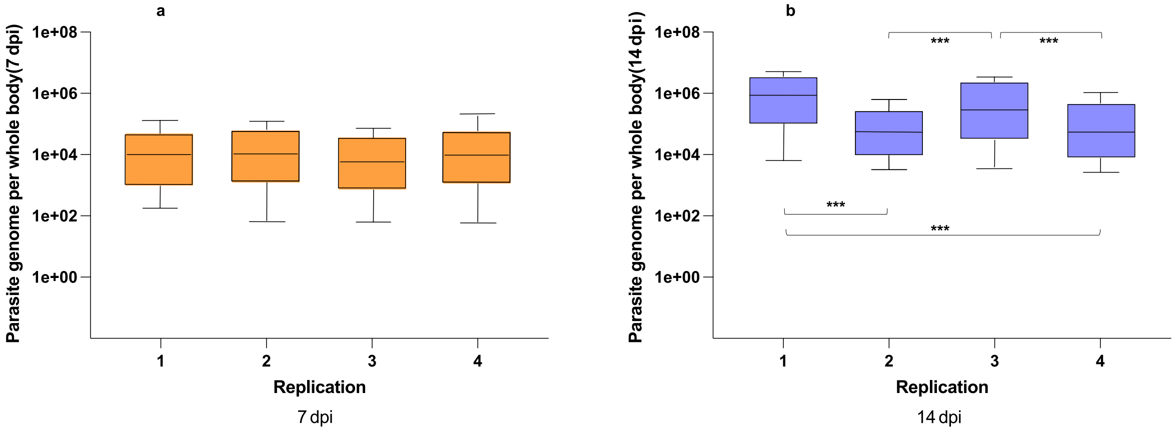

Supplementary Fig. 3.

Quantification of *Plasmodium falciparum* infection load in *Anopheles gambiae* 7 and 14 days post-infection (dpi) for oocysts and sporozoites, respectively. The total number of parasites per whole body of individual mosquitoes was estimated using quantitative PCR. The infection load values are taken from negative binomial model estimations. The median is represented as a line, the box represents the upper and lower quartile range, and the whiskers denote the 95% CI. Comparisons that were statistically significant are shown (***p≤0.001).

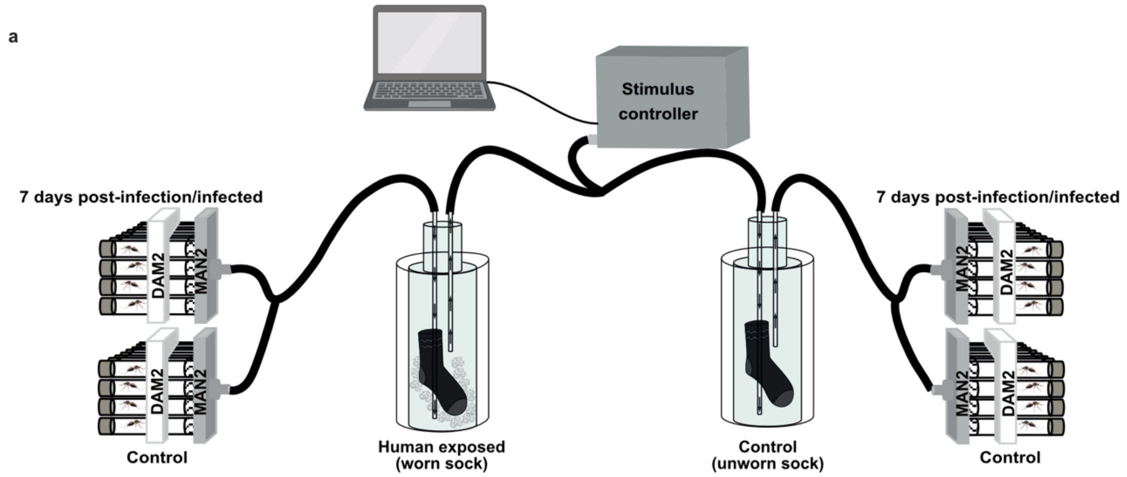

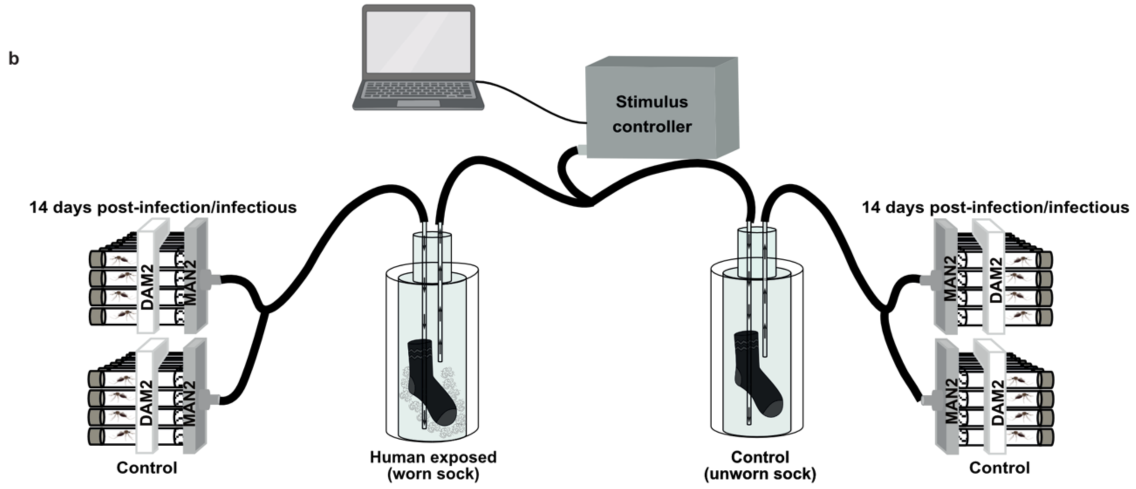

Supplementary Fig. 4.

Schematic design of the locomotor assay. DAM2 assay used to assess the activity of female *Anopheles gambiae*, 7 days post-infection (dpi) and 14 dpi with *P. falciparum* compared to age-matched uninfected females (days control, dc), in the presence (with human odour) or absence of human odour (w/o human odour). Individual locomotor profiles (n = 384), assessed for variation using a redundancy analysis, demonstrated a differential effect of age post-blood meal (The mixed model was build based on the effect of age as a main fixed variable combined with the random effect of the other variables; β-lmer ± SE= age + experimental replication|1).

Supplementary Table 1.

Significant differences in locomotor activity during various informative time periods between age-matched *Anopheles gambiae* females in the presence and absence of human odour. Infected and uninfected females were analysed separately. These results correspond with the dashed brackets in Fig. 1d.

Zeitgeber Time	Condition	*p*–value	Critical value
0 – 11	7 dc without human odour vs. 7 dc with human odour	0.04	−2.11
0 – 11	14 dc without human odour vs. 14 dc with human odour	*p*<0.001	−5.28
0 – 11	7 dpi without human odour vs. 7 dpi with human odour	0.76	0.30
0 – 11	14 dpi without human odour vs. 14 dpi with human odour	0.59	−0.53
11 – 12	7 dc without human odour vs. 7 dc with human odour	*p*<0.001	−6.6
11 – 12	14 dc without human odour vs. 14 dc with human odour	*p*<0.001	5.18
11 – 12	7 dpi without human odour vs. 7 dpi with human odour	0.003	3.11
11 – 12	14 dpi without human odour vs. 14 dpi with human odour	0.001	−3.37
12 – 23	7 dc without human odour vs. 7 dc with human odour	*p*<0.001	15.42
12 – 23	14 dc without human odour vs. 14 dc with human odour	0.01	2.96
12 – 23	7 dpi without human odour vs. 7 dpi with human odour	0.35	−0.92
12 – 23	14 dpi without human odour vs. 14 dpi with human odour	0.89	0.12
23 – 24	7 dc without human odour vs. 7 dc with human odour	0.001	2.69
23 – 24	14 dc without human odour vs. 14 dc with human odour	0.049	−1.87
23 – 24	7 dpi without human odour vs. 7 dpi with human odour	0.27	−1.11
23 – 24	14 dpi without human odour vs. 14 dpi with human odour	0.049	−1.81

Supplementary Table 2.

Significant differences in locomotor activity during various informative time periods across ages (a) and infection stages (b) in *Anopheles gambiae* females in the presence and absence of human odour. These results correspond with the solid brackets and letters in Fig. 1d.

a)

**Zeitgeber Time**	**Condition**	***p*-value**	**Critical value**
0-11	7dc with human odour vs. 14dc with human odour	$p < 0.001$	17.54
0-11	7dpi with human odour vs.14dpi with human odour	0.002	9.35
0-12	7dc with human odour vs. 14dc with human odour	0.005	7.67
0-12	7dpi with human odour vs.14dpi with human odour	$p < 0.001$	20.02
12-23	7dc with human odour vs. 14dc with human odour	$p < 0.001$	15.21
12-23	7dpi with human odour vs.14dpi with human odour	0.001	9.37
23-24	7dc with human odour vs. 14dc with human odour	$p < 0.001$	18.25
23-24	7dpi with human odour vs.14dpi with human odour	$p < 0.001$	15.40
0-11	7dc without human odour vs. 14dc without human odour	$p < 0.001$	27.43
0-11	7dpi without human odour vs. 14dpi without human odour	$p < 0.001$	21.97
0-12	7dc without human odour vs. 14dc without human odour	0.049	4.58
0-12	7dpi without human odour vs. 14dpi without human odour	0.18	1.71
12-23	7dc without human odour vs. 14dc without human odour	0.03	4.33
12-23	7dpi without human odour vs. 14dpi without human odour	0.57	0.31
23-24	7dc without human odour vs. 14dc without human odour	$p < 0.001$	32.32
23-24	7dpi without human odour vs. 14dpi without human odour	0.06	3.49

b)

**Zeitgeber Time**	**Condition**	***p*-value**	**Critical value**
0-11	7dc with human odour vs. 7dpi with human odour	0.001	12.28
0-11	14dc with human odour vs. 14dpi with human odour	0.13	2.26
0-12	7dc with human odour vs. 7dpi with human odour	0.10	2.20
0-12	14dc with human odour vs. 14dpi with human odour	$p < 0.001$	16.06
12-23	7dc with human odour vs. 7dpi with human odour	0.45	0.48
12-23	14dc with human odour vs. 14dpi with human odour	0.001	25.42
23-24	7dc with human odour vs. 7dpi with human odour	0.006	7.29
23-24	14dc with human odour vs. 14dpi with human odour	$p < 0.001$	23.78
0-11	7dc without human odour vs. 7dpi without human odour	0.03	8.49
0-11	14dc without human odour vs. 14dpi without human odour	0.01	5.92
0-12	7dc without human odour vs. 7dpi without human odour	0.31	1.008
0-12	14dc without human odour vs. 14dpi without human odour	0.10	2.68
12-23	7dc without human odour vs. 7dpi without human odour	0.005	7.84
12-23	14dc without human odour vs. 14dpi without human odour	$p < 0.001$	11.87
23-24	7dc without human odour vs. 7dpi without human odour	$p < 0.001$	13.78
23-24	14dc without human odour vs. 14dpi without human odour	0.10	2.20
